# Rise of the Resistance: A Case of Resistant Shigellosis in an Immunocompetent Female Patient

**DOI:** 10.7759/cureus.98272

**Published:** 2025-12-01

**Authors:** Ashley Saito, Jordan Edwards, Adela M Greeley

**Affiliations:** 1 Internal Medicine, University of California, Los Angeles (UCLA) David Geffen School of Medicine, Los Angeles, USA; 2 Hospital Medicine, Greater Los Angeles Veterans Affairs (VA) Healthcare System, Los Angeles, USA; 3 Medicine, Alameda Health System, Highland Hospital, Oakland, USA

**Keywords:** antimicrobial resistance, gastroenteritis, shigella, stool culture, symptoms of enteritis

## Abstract

Shigellosisis a common enteric infection with updated empiric treatment guidance due to the emergence of resistant isolates. Herein, we describe a case of resistant shigellosis in an immunocompetent female with no recent travel. The patient initially started ciprofloxacin, but due to ongoing gastrointestinal symptoms and intermediate resistance to fluoroquinolones on stool culture, she was later switched to azithromycin. We believe this case demonstrates the looming threat of multidrug-resistant *Shigella*, even among those without risk factors or recent antibiotic use. This case also highlights the importance of stool culture susceptibility testing.

## Introduction

Shigellosis is a highly infectious, often self-limited enteric illness characterized by bloody stools, fever, and anorexia [1]. Empiric treatment is recommended in select patients to reduce symptom duration and disease spread. While fluoroquinolones are the recommended treatment by the World Health Organization (WHO), there are increasing reports of resistant *Shigella* isolates worldwide [2,3]. Empiric antimicrobial treatment is no longer guaranteed to result in clinical improvement. Close follow-up on susceptibility testing is recommended whenever possible to ensure an appropriate antibiotic was selected. Here, we describe a case of an immunocompetent female with symptomatic shigellosis who was found to have a resistant isolate identified on stool culture testing. This warranted a change in her outpatient antimicrobial regimen to ensure effective treatment and symptom resolution.

## Case presentation

A 37-year-old female with insulin-dependent type 2 diabetes mellitus presented to the Emergency Department (ED) for evaluation of acute abdominal pain, fever, and chills. Her symptoms developed suddenly the evening prior to admission. She denied recent travel or sick contacts. She reported nausea without emesis and normal bowel movements during this time. Her menstrual cycles were regular. She denied any new sexual partners or history of sexually transmitted infections.

On arrival in the ED, her vital signs were notable for a temperature of 103°F, a heart rate of 140 beats per minute, and a blood pressure of 116/73 mmHg. A physical exam revealed tenderness in the lower abdomen without guarding, rebound, or rigidity. Laboratory workup was notable for an elevated white blood cell count with neutrophilic predominance. Her sodium and bicarbonate were both low, but her anion gap was just within normal range at 12 mmol/L. Her creatinine was normal, but the glucose was elevated (Table 1). Her serum lactate was also elevated. Urinalysis showed glucosuria with trace ketones but no nitrites, leukocyte esterase, or bacteria. Her urine pregnancy test was negative.

**Table 1:** Laboratory results from the day of hospital admission

Test	Value	Reference Range
Complete blood count		
White blood cell count	12.98×10^3^/µL	4.0–11.0×10^3^/µL
% Neutrophils	82.7%	40–60%
Hemoglobin	14.7 g/dL	Female: 12.0–15.5 g/dL
Platelet count	233×10^3^/µL	150–400×10^3^/µL
Metabolic panel		
Sodium	131 mmol/L	135–146 mmol/L
Potassium	3.2 mmol/L	3.6–5.3 mmol/L
Chloride	100 mmol/L	96–106 mmol/L
Bicarbonate	19 mmol/L	20–30 mmol/L
Urea nitrogen	12 mg/dL	7–22 mg/dL
Creatinine	0.67 mg/dL	0.60–1.30 mg/dL
Glucose	336 mg/dL	65–99 mg/dL
Lactate	2.48 mmol/L	0.5–2.2 mmol/L

**Figure 1:**
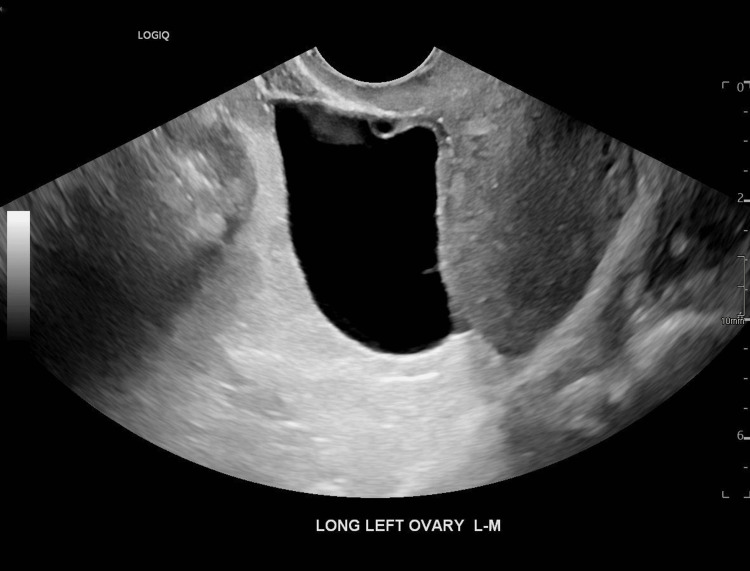
Transvaginal ultrasound demonstrating a 5 cm left ovarian cyst in long-axis view

**Figure 2:**
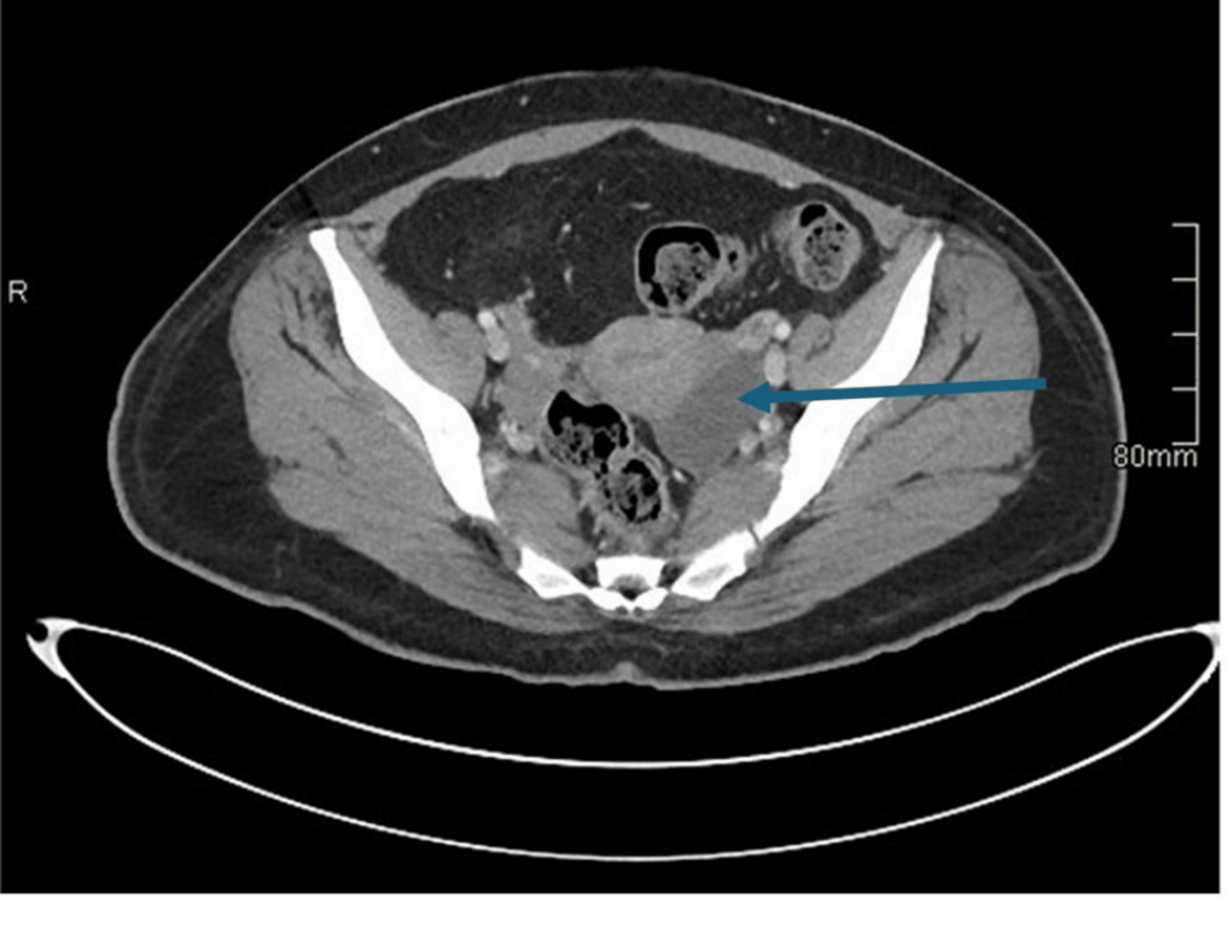
CT of the abdomen and pelvis with contrast redemonstrating her left ovarian cyst (blue arrow) in an axial plane

Transvaginal ultrasound (Figure 1) and computed tomography (CT) of the abdomen and pelvis (Figure 2) noted a 5 cm left ovarian cyst without signs of torsion. No other significant findings were noted. COVID and influenza swabs were negative. She received a total of 6 L of crystalloid fluid for volume resuscitation, antipyretics, and intravenous ceftriaxone. She was admitted to the general medicine service for further evaluation and care of sepsis presumed secondary to a gastrointestinal source.

On hospital day two, the patient reported new watery, non-bloody diarrhea. A stool culture was collected and returned positive for *Shigella*. The patient was started on oral ciprofloxacin, and the Department of Public Health was notified. The following day, her fever, chills, and leukocytosis resolved, and she was discharged home. Following hospital discharge, the susceptibilities of her *Shigella* isolate were determined (Table 2). The patient's *Shigella* isolate demonstrated intermediate resistance to fluoroquinolones and resistance to ampicillin and trimethoprim-sulfamethoxazole. The patient was called and reported ongoing gastrointestinal symptoms, despite improvements in her fever and leukocytosis at the time of hospital discharge. Infectious Diseases was consulted, and due to her ongoing symptoms, ciprofloxacin was changed to azithromycin 500 mg once daily for three days. At the time of her outpatient provider follow-up, the patient reported complete resolution of her symptoms.

**Table 2:** Shigella susceptibility report *Ceftriaxone and aztreonam susceptibilities were not initially reported but were requested following the Infectious Diseases consultation. MIC: minimum inhibitory concentration

Antibiotic	MIC	Interpretation
Ampicillin	> 32.0 mcg/mL	Resistant
Aztreonam*	< 1 mcg/mL	Susceptible
Ceftriaxone*	< 1 mcg/mL	Susceptible
Ciprofloxacin	0.5 mcg/mL	Intermediate
Trimethoprim/sulfamethoxazole	> 320 mcg/mL	Resistant

## Discussion

*Shigella *species are highly virulent, gram-negative enteric pathogens that remain a leading cause of diarrheal illness worldwide [4]. Transmission occurs primarily via the fecal-oral route, often through contaminated water sources or poor sanitation in developing countries [1]. In the United States, shigellosis has historically been a self-limited diarrheal illness seen among young children (ages one to seven years) [2,3]. More recently, cases of multidrug-resistant shigellosis have been reported among men who have sex with men (MSM), people experiencing homelessness, and returning international travelers [2]. The emergence of resistant strains has reduced the number of antibiotics available to treat shigellosis and demands closer clinician follow-up for susceptibility testing. Here, we describe a case of resistant shigellosis in an immunocompetent female without any of the aforementioned risk factors. We believe this case highlights the threat of multidrug-resistant *Shigella *even for those without risk factors or recent antibiotic use.

*Shigella *species are part of the larger *Enterobacteriaceae* family and are comprised of four pathogenic species: *Shigella sonnei*, *Shigella dysenteriae*, *Shigella flexneri*, and *Shigella boydii* [5]. *Shigella *is spread by direct contact with an infected person or by consumption of contaminated food or water [1]. Compared to other enteric pathogens, *Shigella *has a very low infectious dose (as few as 10 organisms) that facilitates its rapid spread [2]. Classic symptoms of shigellosis include watery or bloody diarrhea, abdominal cramping, anorexia, and fever [4]. Severe cases may present with toxic megacolon or hemolytic uremic syndrome (HUS) [1]. For many, the illness is self-limited, and a complete recovery is seen within 7-10 days [4].

Oral rehydration is the mainstay of treatment for all patients with suspected shigellosis [3,4]. Anti-diarrheal agents should be avoided due to their potential to delay excretion of the Shiga toxin and prolong clinical symptoms [3]. Antimicrobial treatment is reserved for those with immunocompromising conditions and/or severe symptoms such as bacteremia, intestinal or extraintestinal complications (i.e., HUS), or dehydration requiring hospitalization [4]. The goal of antimicrobial treatment is to reduce symptom duration and, in select cases, to reduce the spread of infection to other individuals.

Empiric treatment of shigellosis has changed in recent years due to the emergence of resistant *Shigella *strains [2]. While beta-lactam antibiotics such as ampicillin were previously first-line agents, the WHO now recommends ciprofloxacin [4]. This recommendation is a result of widespread ampicillin resistance, with one study of 1,376 *Shigella *strains from 2000 to 2010 revealing that 74% were resistant to ampicillin [5]. However, the increasing use of fluoroquinolones across healthcare settings may already be diminishing their efficacy.

Fluoroquinolone-resistant *Shigella* isolates have steadily increased in the United States over time. In 2016, the Centers for Disease Control and Prevention (CDC) reported that 2% of all Shigella isolates were resistant to ciprofloxacin [6]. In 2020, the rate of ciprofloxacin resistance rose to 18% [6]. The rising rates of fluoroquinolone resistance mirror what is seen on a global level. In 2023, the WHO found that 29.7% of all *Shigella* strains worldwide demonstrated ciprofloxacin resistance [7].

In 2017, the CDC issued an advisory warning that *Shigella *isolates categorized as "susceptible" to fluoroquinolones were found to harbor multiple resistance mechanisms that predisposed them to treatment failure [2]. This advisory resulted in the Clinical and Laboratory Standards Institute (CLSI) increasing the minimum inhibitory concentration (MIC) cutoff for *Shigella *isolates against fluoroquinolones. As reflected in our patient's case, the CLSI now categorizes *Shigella* isolates with MIC values between 0.12 and 1.0 mcg/mL as intermediate susceptibility, whereas previously values in this range were considered susceptible [8]. *Shigella *isolates with no resistance mechanisms to fluoroquinolones typically have an MIC of less than 0.015 mcg/mL and are increasingly rare [8]. As MICs for fluoroquinolones continue to rise, clinicians must be vigilant in following up on susceptibility reports to ensure the antibiotics prescribed are effective in treating their patients.

Mechanisms of fluoroquinolone resistance among *Shigella *isolates are varied but noteworthy for chromosomal mutations acquired from antimicrobial exposure and those acquired even without fluoroquinolone exposure [9]. Mutations in bacterial DNA gyrase (gyrA) and topoisomerase IV (parC) in the quinolone-determining regions (QRDRs) play a key role in increased *Shigella* resistance following exposure to fluoroquinolones [10]. *Shigella* isolates that develop resistance genes without prior antibiotic exposure utilize plasmid-mediated quinolone resistance (PMQR) mechanisms encoded within mobile genetic elements [9]. While chromosomal mutations in gyrA and parC are important contributors to rising rates of fluoroquinolone resistance worldwide, the PMQR mechanism is responsible for transferable fluoroquinolone resistance to non-exposed *Shigella *isolates. This PMQR mechanism may explain why our patient's *Shigella *isolate displayed intermediate susceptibility to ciprofloxacin despite no recent antimicrobial exposure.

In the face of rising resistance patterns, WHO recommends ceftriaxone as a second-line agent and azithromycin as an alternate agent in adults, although some laboratories do not routinely evaluate *Shigella *isolates for azithromycin susceptibility [3,4]. The duration of treatment depends on the selected antimicrobial. Fluoroquinolones and azithromycin are typically dosed for three days; beta-lactams for five days. If appropriate antibiotics are selected, most patients experience symptomatic improvement within the following 48 hours [4].

It is critical to remember that empiric antimicrobial treatment is reserved for patients who are immunocompromised or experiencing severe symptoms (i.e., bacteremia or dehydration requiring hospitalization) from presumed bacterial gastroenteritis [3,4]. Even when *Shigella *is isolated, an immunocompetent patient without severe symptoms often improves spontaneously with supportive measures alone. Extensively drug-resistant (XDR) isolates of *Shigella *are now being reported, with XDR strains being defined as those resistant to ampicillin, azithromycin, ciprofloxacin, ceftriaxone, and trimethoprim-sulfamethoxazole (TMP-SMX) [2]. To date, there are no best practices to inform clinicians about the management of XDR *Shigella*. With this looming threat on the horizon, it is important for us as clinicians to practice antimicrobial stewardship and follow up on susceptibility testing. These simple measures may help combat the rise of resistant isolates and preserve our antimicrobial treatment options for those who need them most.

## Conclusions

Increasingly resistant isolates of *Shigella *have emerged, particularly among returning international travelers, unhoused individuals, and MSM. However, resistant cases are now also being observed in patients without these risk factors. While shigellosis is often a self-limited enteric illness, antimicrobial treatment is indicated for those with immunocompromising conditions and/or severe symptoms requiring hospitalization. Ciprofloxacin remains the first-line treatment for those with severe shigellosis. When a resistant isolate is identified, the WHO recommends ceftriaxone as a second-line agent and azithromycin as an alternate agent in adults.
